# Diversity and Functionality of Culturable Endophytic Bacterial Communities in Chickpea Plants

**DOI:** 10.3390/plants8020042

**Published:** 2019-02-14

**Authors:** Clarisse Brígido, Sakshi Singh, Esther Menéndez, Maria J. Tavares, Bernard R. Glick, Maria do Rosário Félix, Solange Oliveira, Mário Carvalho

**Affiliations:** 1 ICAAM—Instituto de Ciências Agrárias e Ambientais Mediterrânicas, Universidade de Évora, Pólo da Mitra, Ap. 94, 7002-554 Évora, Portugal; esthermenendez@uevora.pt (E.M.); tavares.mja@gmail.com (M.J.T.); mrff@uevora.pt (M.d.R.F.); mjc@uevora.pt (M.C.); 2 IIFA—Instituto de Investigação e Formação Avançada, Universidade de Évora, Ap. 94, 7002-554 Évora, Portugal; 3 Amity Institute of Microbial Technology, 4th Floor, E-3 BlockSector, Sector 125, Noida, Uttar Pradesh 201313, Índia; sakchaudhary@gmail.com; 4 Department of Biology, University of Waterloo, Waterloo, ON N2L 3G1, Canada; glick@uwaterloo.ca

**Keywords:** endophytes, *Cicer arietinum*, plant growth-promoting bacteria, mechanisms, rhizobia inoculation, manganese, salinity

## Abstract

The aims of this study were to isolate, identify and characterize culturable endophytic bacteria from chickpea (*Cicer arietinum* L.) roots grown in different soils. In addition, the effects of rhizobial inoculation, soil and stress on the functionality of those culturable endophytic bacterial communities were also investigated. Phylogenetic analysis based on partial 16S rRNA gene sequences revealed that the endophytic bacteria isolated in this work belong to the phyla Proteobacteria, Firmicutes and Actinobacteria, with *Enterobacter* and *Pseudomonas* being the most frequently observed genera. Production of indoleacetic acid and ammonia were the most widespread plant growth-promoting features, while antifungal activity was relatively rare among the isolates. Despite the fact that the majority of bacterial endophytes were salt- and Mn-tolerant, the isolates obtained from soil with Mn toxicity were generally more Mn-tolerant than those obtained from the same soil amended with dolomitic limestone. Several associations between an isolate’s genus and specific plant growth-promoting mechanisms were observed. The data suggest that soil strongly impacts the Mn tolerance of endophytic bacterial communities present in chickpea roots while rhizobial inoculation induces significant changes in terms of isolates’ plant growth-promoting abilities. In addition, this study also revealed chickpea-associated endophytic bacteria that could be exploited as sources with potential application in agriculture.

## 1. Introduction

Plants, including legumes, are normally colonized by a wide range of different microorganisms [1]. A subset of those microbes consists of endophytic bacteria, bacteria that colonize the internal tissues of a plant without any apparent sign of infection or negative effects on the host plant [2], and represents a widespread and ancient relationship [3]. However, few associations between plants and endophytes have been studied in detail, with the legume-rhizobia association being the exception. These bacteria can promote plant growth in a variety of ways. For instance, they can improve plant growth by increasing the availability and uptake of nutrients [4,5], by fixing nitrogen [6,7], by producing phytohormones [4,8,9], by modulating plant ethylene levels [10] and by suppressing plant diseases [11,12].

It is generally accepted that multiple factors, such as different plant tissues and phenotypes, season and soil conditions, have impacts on the communities of bacterial species present within the host plants [13,14,15,16]. For instance, some studies conducted in soybean showed that plant growth stage and tissue, treatment with the herbicide glyphosate, nodulation phenotype and nitrogen level had different effects on the diversity and taxonomic composition of the endophytic bacterial community [8,16,17,18]. However, whether these changes have specific consequences for plant growth and health remains unknown.

It is now a common agricultural practice to use legume seeds inoculated with compatible rhizobia to provide sufficient numbers of viable and effective bacteria for rapid and efficient colonization of the host rhizosphere [19], in order to supply nitrogen to legume tissues [20]. However, it is still largely unknown how this treatment affects the soil microbial composition, and consequently, the soil enzymes and the endophytic bacterial community within plant tissues.

Although it is known that different factors affect the diversity of bacterial communities associated with different plants, little is known about the multifunctionality of these communities, especially non-rhizobial endophytic bacteria in legumes. The effects of rhizobial inoculation as well as soil conditions on the chickpea endophytic bacterial communities have not yet been studied. Given that chickpea (*Cicer arietinum* L.) is one of the most important grain legumes in the world, and considering the potential of endophytic bacteria on legume growth and health, studies on those interactions should contribute to a better understanding of how these interactions are affected by soil conditions and by common agricultural practices, such as seed inoculation with rhizobia. In this work, we investigated the diversity and the multifunctionality of culturable endophytic bacteria isolated from chickpea roots and determined whether rhizobia inoculation, soil and stress influence those communities. Our data reveal several endophytic bacteria associated with chickpea that could be exploited as sources with potential application in agriculture. Furthermore, although preliminary, this study suggests that different variables shape the functionality of endophytic bacterial communities; these prominently include the soil origin (including aboveground diversity) and the presence or absence of rhizobial inoculation.

## 2. Results

### 2.1. Isolation and Identification of Bacterial Endophytes from Chickpea Roots

**Figure 1:**
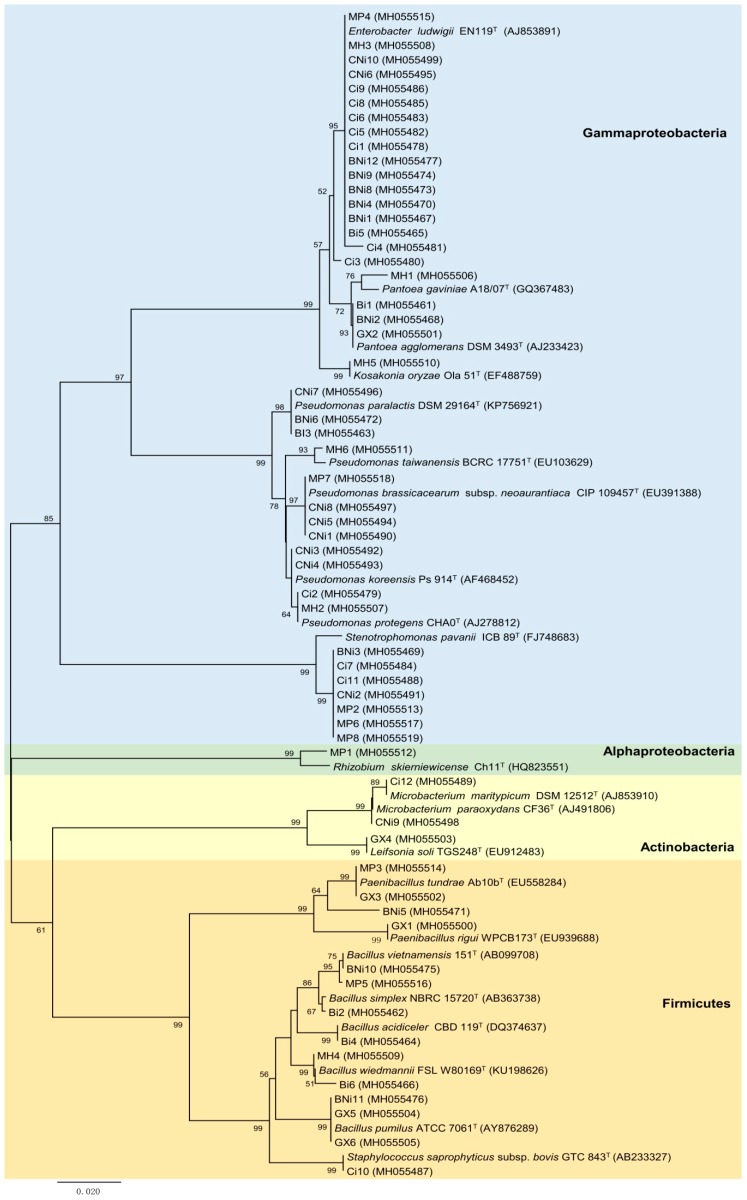
Neighbor-joining phylogenetic tree based on the partial sequence of the 16S rRNA gene of bacterial isolates from chickpea roots and their related type strains. The evolutionary distances were computed using the Kimura 2-parameter method [21]. Nodes were maintained when the maximum-likelihood algorithm was applied. There are a total of 521 positions in the final dataset. Bootstrap values are given at branch nodes and are based on 1000 replicates (values higher than 50% are indicated). Accession numbers are provided in parentheses.

A total of 59 culturable endophytic bacteria were isolated from chickpea roots (Table 1). Based on their partial 16S rDNA nucleotide sequences, isolates were classified into 3 phyla: Proteobacteria, Firmicutes and Actinobacteria (Figure 1). Proteobacteria was the most abundant phylum, accounting for ~71% of total isolates. All Proteobacteria isolates belong to class Gammaproteobacteria with the exception of one isolate, a *Rhizobium* sp. strain MP1, which belongs to the class Alphaproteobacteria. Within the Gammaproteobacteria, the family *Enterobacteriaceae* was the most represented, comprising 22 isolates, including the genera *Kosakonia*, *Klebsiella*, *Pantoea* and *Enterobacter*, followed by the families *Pseudomonadaceae* and *Xanthomonadaceae*, with 13 and 6 isolates, respectively (Figure 1). Moreover, the genera *Leifsonia*, *Staphylococcus*, *Klebsiella*, *Kosakonia* and *Rhizobium* showed frequencies lower than 2% while *Bacillus*, *Stenotrophomonas*, *Pseudomonas* and *Enterobacter* were the most prevalent genera, all with frequencies higher than 10%.

Although the low number of isolates obtained per treatment did not allow an in-depth analysis of the effects of the soil, inoculation with rhizobia and stress, in the diversity and endophytic bacteria composition, some differences were observed. For instance, despite the high frequency of *Pseudomonas* and *Enterobacter* genera, these genera were not commonly found in all soil samples (Table 2). In fact, just the genus *Bacillus* was generally identified in all soils if we consider only the four original soils, namely, Gaxa, Malheiros, Monte da Pedra and Herdade da Mitra without any treatment, i.e., addition of dolomitic limestone and seed inoculation with *Mesorhizobium*. On the other hand, the genus *Kosakonia* was only present in the MH treatment whereas the genera *Rhizobium* and *Leifsonia* were exclusively found in the MP and Gaxa treatments, respectively. Also differences in the endophytic bacterial community composition present in chickpea roots grown in the Herdade da Mitra soil were observed when this soil was amended with dolomitic limestone. Although the presence of the genera *Enterobacter* and *Pseudomonas* was detected in both treatments, the frequency of the genus *Pseudomonas* increased after the soil amendment while the genus *Enterobacter* decreased (Figure 1, Table S1). Moreover, isolates belonging to the genera *Paenibacillus* and *Pantoea* were only found in chickpea plants grown in the Herdade da Mitra soil without dolomitic limestone whereas isolates assigned to the genus *Microbacterium* was only found in the amended soil. In contrast, although differences in the frequency of a specific genus were observed, no significant changes were observed between the endophytic bacteria composition found in chickpea plants grown in the Herdade da Mitra soil with and without rhizobial inoculation. Similarly, despite the fact that the presence of the genera *Staphylococcus* and *Klebsiella* was only detected in the dolomitic limestone amendment soil with rhizobial inoculation, the effect of rhizobium inoculation on the endophytic bacteria composition in that soil did not change greatly.

### 2.2. Evaluation of Bacterial Endophytes Potential for Plant Growth Promotion and Cellulase Production

**Figure 2:**
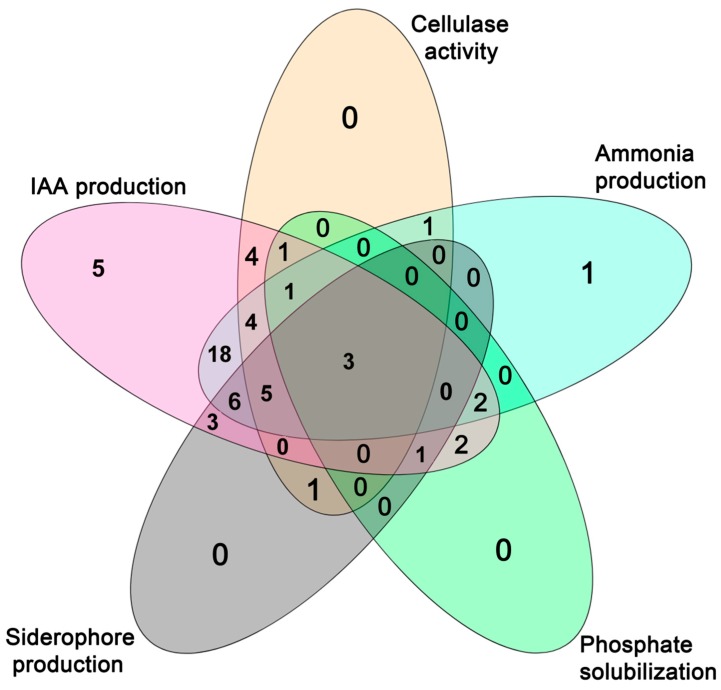
Venn diagram showing the number of isolates possessing each of the different plant growth-promoting characteristics, namely, phosphate solubilization, indoleacetic acid synthesis, siderophore and ammonia production and cellulase activity. Note that not determined plant growth-promoting characteristics in some strains were considered as absent in this graphic.

**Figure 3:**
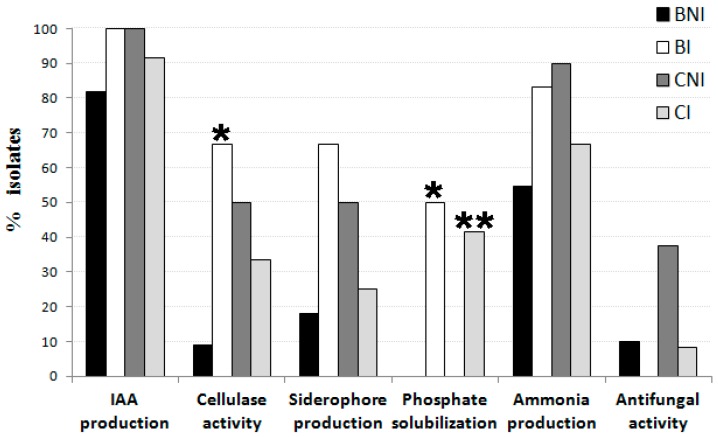
Proportion of isolates possessing different plant growth-promoting traits and cellulase activity from Herdade da Mitra soil (BNI) with rhizobial inoculation (BI) or with correction with dolomitic limestone (CNI) and with rhizobial inoculation (CI). Significant proportions detected with Fisher’s exact test between BNI and BI (*) or CNI and CI (**).

The bacterial endophytes isolated from chickpea roots were evaluated for their cellulase activity and plant growth promotion potential, namely, indole-3-acetic acid (IAA), siderophore and ammonia production, phosphate solubilization, and antifungal activity (Table S1). Twenty of the (33.9%) bacterial endophytes showed positive results for cellulase activity (Figure 2, Table S1). Moreover, an association between the levels of cellulase activity and the isolate’s genera was found (*P* < 0.05). Most of the isolates belonging to the genera *Stenotrophomonas* and *Enterobacter* did not display any cellulase activity while the highest cellulase activity was detected in isolates assigned to *Bacillus*, *Pseudomonas* and *Paenibacillus* genera. Although the proportion of cellulase-producing isolates in the treatments CNI, CI and BI was higher than that in the Herdade da Mitra soil without amendement and without rhizobial inoculation (BNI), only the the proportion of those isolated in the CI treatment was significantly higher (Figure 3).

Most of the isolates (>93%) were able to synthesize IAA-like molecules when grown in minimal liquid medium supplemented with 250 µg·mL^−1^ of tryptophan (Figure 2, Table S1); however, only 40.6% of them were able to produce more than 10 µg·mL^−1^ of IAA-like molecules. Similar to what was observed for cellulase activity, the levels of IAA production between genera were also significantly different (*P* < 0.001). Isolates from *Bacillus*, *Paenibacillus*, *Pseudomonas* and *Stenotrophomonas* showed only a low level of IAA production while a high level of IAA production was displayed by isolates belonging to the genus *Enterobacter*. Albeit no statistically significant difference between the means of IAA produced by the isolates obtained from each treatment was observed, the average amount of IAA produced by different isolates varied greatly between soil treatments. For instance, the highest mean IAA production (≥40 µg·mL^−1^) was achieved by endophytic bacteria isolated from the plants grown in the treatment CI and BI (Herdade da Mitra soil with and without dolomitic limestone, and seed inoculation with *Mesorhizobium ciceri* LMS-1) while the lowest mean IAA production (2 µg·mL^−1^) was produced by the isolates obtained from treatment GX (Gaxa treatment) (Table S1). Curiously, the bacterial isolates obtained from plants grown in the treatment CNI (Herdade da Mitra soil plus dolomitic limestone and non-inoculation) registered an IAA production average that was considerably lower than that found in treatment BNI (Herdade da Mitra soil and non-inoculation). This result suggests that soil amendment with dolomitic limestone per se decreased the prevalence of endophytic bacteria that produced a high level of IAA within chickpea roots, while rhizobial inoculation contributed to an increase in the presence of these endophytes in plants grown in soil without limestone amendment.

Similar to IAA production, a high proportion (69.5%) of the endophytic bacterial isolates tested showed the ability to produce ammonia (Figure 2, Table S1), revealing that this ability is also a common plant growth-promoting feature among these isolates independent of the treatment. In contrast, only 33.3% and 17.5% of the tested bacterial endophytes isolates showed positive results for siderophore production and phosphate solubilization, respectively (Figure 2, Table S1). Notably, significant associations between the isolate’s affiliation at the genus level and its ability to produce ammonia (*P* < 0.05) and to solubilize phosphate (*P* < 0.05) were found. For instance, almost all isolates from the genera *Bacillus*, *Enterobacter* and *Pseudomonas* produced ammonia while no isolates assigned to the genera *Leifsonia*, *Paenibacillus* and *Staphylococcus* possessed this trait. Likewise, all isolates belonging to the genera *Klebsiella*, *Leifsonia*, *Kosakonia* and *Staphylococcus* were able to solubilize phosphate whereas the majority of the isolates belonging to the other genera were not able to do so. Curiously, almost all endophytic bacteria possessing the ability to solubilize phosphate were isolated from Herdade da Mitra soil either with or without a limestone amendment, but exclusively with *Mesorhizobium* inoculation (BI and CI treatments) (Figure 3, Table S1). The latter observation suggests that the presence of a *Mesorhizobium* strain somehow influenced the interaction between chickpea plants and phosphate-solubilizing endophytic bacteria. Only seven isolates, namely, *Paenibacillus* sp. BNI-5, *Pseudomonas* sp. CI-2, *Stenotrophomonas* sp. CNI-2, *Pseudomonas* sp. CNI-3, *Pseudomonas* sp. CNI-4, *Pseudomonas* sp. MH2 and *Bacillus* sp. MH4, showed antifungal activity against *Fusarium oxysporum* f. sp. ciceri (Table S1). No association was found between isolates’ antifungal activity and soil origin or genus affiliation, or any other specific plant growth-promoting trait.

**Figure 4:**
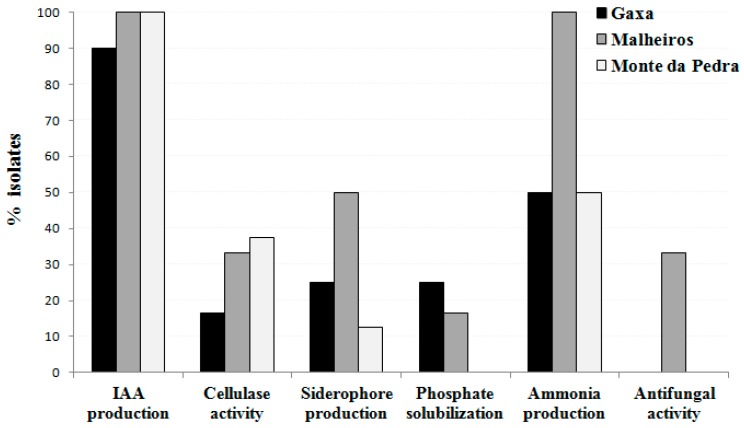
Proportion of bacterial isolates possessing different plant growth-promoting traits and cellulase activity from Gaxa (GX), Malheiros (MH) and Monte da Pedra (MP) soils.

The majority (77.7%) of the endophytic bacteria possess two or more plant growth-promoting features, and 35.6% of them have three or more of the plant growth-promoting traits tested. The isolates obtained from the GX and MP treatments presented the fewest plant growth-promoting traits (Figure 4, Table S1). On the other hand, the majority of the isolates that exhibit more plant growth-promoting features were obtained from chickpea plants grown in Herdade da Mitra soil samples, regardless as to whether or not those had Mn toxicity. This explains the association found between the number of multi-trait isolates and the soil treatments (*P* < 0.05).

### 2.3. Evaluation of Endophytic Bacterial Tolerance to Salt and Manganese

**Figure 5:**
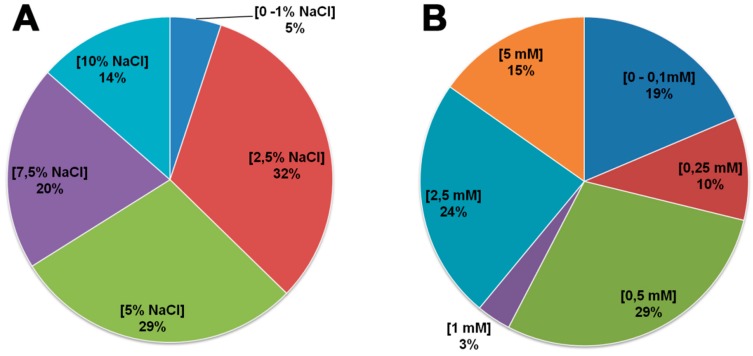
Percentage of bacterial isolates tolerant to either (**A**) salt (% NaCl) or (**B**) Mn (mM MnSO4).

Nearly all of the endophytic bacterial isolates showed tolerance to high salt concentrations (Figure 5a), with *Pseudomonas* sp. CI-11, *Paenibacillus* sp. GX1 and *Bacillus* sp. GX5 isolates being the exception (i.e., growth inhibition at ≥2.5% NaCl). Similarly, 71% of the endophytic bacterial isolates tolerated high manganese concentrations (≥0.5 mM MnSO_4_) (Figure 5b).

**Figure 6:**
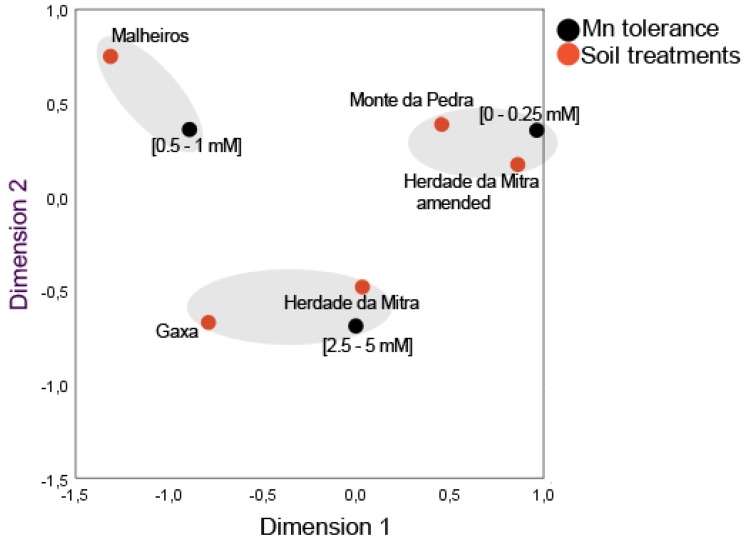
CA biplot of the relationship between the isolate’s tolerance to Mn and their soil origin or treatment. Data from Herdade da Mitra soils with and without addition of dolomitic limestone and exclusively without seed inoculation.

Moreover, an isolate’s ability to tolerate salt or manganese was associated with its affiliation at the order level (*P* < 0.01). That is, isolates belonging to the orders Bacillales and Enterobacteriales were highly salt-tolerant whereas isolates assigned to Pseudomonadales and Xanthomonadales orders were more sensitive to salt stress. Pseudomonadales, Bacillales and Actinomycetales isolates showed sensitivity to manganese while Enterobacteriales isolates were highly Mn-tolerant. In addition, the isolates obtained from Herdade da Mitra soil were generally found to be more Mn-tolerant than those obtained from the other soils (χ^2^ = 23.950; d.f. = 12; *P* < 0.05), and the addition to that soil of dolomitic limestone resulted in the isolation of a higher number of Mn-sensitive isolates (χ^2^ = 9.404; d.f. = 3; *P* < 0.05). In fact, a correspondence analyses (CA) reinforced the previous observation, revealing that isolate’s tolerance to Mn was associated with soil origin (Figure 6). Moreover, the addition of dolomitic limestone to the Herdade da Mitra soil contributed to an increase of the presence of Mn-sensitive isolates in chickpea roots grown in that soil. 

## 3. Discussion

Besides the typical nitrogen-fixing endosymbionts, collectively named as rhizobia, that legume plants harbor inside their root nodules, other endophytic bacteria are usually found within different legume tissues. Although several previous studies have indicated that some of these bacteria are able to promote plant growth and health [22], few reports have focused their attention on the symbiotic or endophytic bacteria that colonize legumes roots. Moreover, the question arises as to what are the variables that determine the diversity and composition of endophytic bacterial communities and what are the key effects on plant fitness.

In this study, the endophytic bacterial isolates were assigned to 12 different genera belonging to three phyla: *Proteobacteria, Firmicutes* and *Actinobacteria*. This result is consistent with other studies where culture-dependent methods were used [23,24,25]. It should be noted that the culture-based method used in this work excludes a portion of the slow-growing and non-culturable endophytic bacteria; therefore, a spectrum of the “true” diversity of endophytic bacteria in chickpea roots could be revealed using DNA-based approaches [26]. Nevertheless, our data reveal a similar diversity pattern to the one obtained from the clover root endosphere, where 84% of the total sequences are represented by Proteobacteria and ~11% correspond to Actinobacteria and Firmicutes [27]. *Enterobacter* and *Pseudomonas* were the most common genera among the chickpea roots followed by *Bacillus*, *Stenotrophomonas*, *Paenibacillus* and *Pantoea*. On the other hand, *Staphylococcus*, *Rhizobium*, *Leifsonia*, *Kosakonia* and *Klebsiella* genera were the least common genera observed. Nevertheless, all these bacterial genera have been identified as endophytes from different plants [28,29,30,31,32].

Despite the limitations of culture-based methods for analyzing microbial diversity [26,33], these methods allow the isolation of culturable bacteria for functional analysis or for obtaining their benefits for agricultural applications [34,35]. In addition, the characterization of the multifunctionality of culturable microbes may also contribute to a better understanding of the function of microbial communities living in close association with plants, as is the case for endophytic bacteria. In terms of plant growth-promoting features, most of the endophytic bacteria possess two or more plant growth characteristics and a high proportion of them were obtained from chickpea plant roots grown at the Herdade da Mitra site. This result may be due to the fact that this soil contained a diverse mixture of natural plants contrary to the other sites where a monoculture was grown. In fact, the literature indicates that the most diversified model ecosystems have a greater number of functionalities compared to less diversified model ecosystems [36]. Recently, the study conducted by Wagg et al. [37] revealed that ecosystem functions are closely related to soil microbial biodiversity, suggesting that the composition of soil communities is the key factor in regulating ecosystem functioning. In fact, the functioning of plant communities is influenced by the presence and diversity of microorganisms in the subsoil, namely, fungi and bacteria, which affect nutrient acquisition capacities and resistance to stress conditions by plants [37,38,39]. Therefore, it appears that the presence of a diverse plant community along with no addition of inputs associated with conventional agriculture in this soil contributed to the multifunctionality of the soil microorganisms, such as the microbe subset studied herein. In addition, other variables, such as the cultivation history and agricultural practices, cannot be disregarded. Indeed, cultivation history was previously determined as an important driver of endophytic colonization in maize plants [40], and the diversity of endophytic bacteria was significantly affected by organic and conventional practices [34]. Therefore, variables that induce changes in the diversity of endophytic bacterial communities may consequently alter the functionality of those communities.

Indoleacetic acid and ammonia production were the most common plant growth-promoting traits found in this study. While one study found a high occurrence of IAA-producing bacteria in the aboveground plant parts [41], other studies have revealed that this trait is very common among bacteria with endophytic behavior [8,42,43], including rhizobia [44]. In addition to the known role of IAA in directly promoting plant growth and development, microbial IAA has also been reported to act as a signaling molecule in several plant-microorganism interactions [45]. The high percentage of bacterial isolates found in this study that are able to produce ammonia is in agreement with the results of Szilagyi-Zecchini et al. [46]. Ammonia production can provide a portion of the nitrogen demand of the host plant [47,48].

Bacterial endophytes may also secrete siderophores and solubilize phosphorus in soil while interacting with host plants [49], where siderophores chelate iron from the environment for use by microbial and plant cells and phosphate solubilization provides phosphorus for plants to absorb [50]. Although phosphate solubilization and siderophore production contribute to an increased nutrient uptake by the host plant, only a few endophytic bacterial isolates possess these abilities. Several reports have shown that some endophytic bacteria also have the ability to solubilize inorganic phosphorus [5,44,51]. However, it is more common to find the ability to solubilize inorganic phosphate among rhizospheric bacteria [52]. Surprisingly, the treatments with inoculated chickpea roots reveal a significantly higher proportion of phosphate-solubilizing isolates than those without *Mesorhizobium* inoculation. A similar effect regarding the proportion of cellulase-producing isolates between CI and CNI treatments was observed. It may be possible that the presence of a *Mesorhizobium* strain may alter the plant-soil-bacteria network, thereby selecting for phosphate-solubilizing or cellulase-producing endophytic bacteria under specific conditions.

Although relatively few of the bacteria isolated in this study were able to synthesize siderophores, most of the isolates with this ability were from plants grown on soil with excessive levels of manganese. In addition to the canonical role of siderophores in scavenging insoluble iron [53], bacterial siderophores can also bind to other non-iron metals [54] reducing those free toxic metal concentrations in the environment [55]. The data presented here agree with the observations of Hesse et al. [56], where the proportion of siderophore-producing bacterial taxa was reported to increase along a natural heavy metal gradient.

One third of the endophytic bacterial isolates present cellulase activity on CMC plates. Cellulase-producing bacteria have been isolated from a wide variety of sources. This activity is highly related to an isolate’s entry and spread within plant tissues [57], since enzymes such as cellulases, xylanases, pectinases, and endoglucanases are used to modify the plant cell wall enabling endophytes to enter and colonize [57,58,59]. This notwithstanding, many other studies point to a situation where natural cracks at the lateral root emergence site are the most common entry sites for endophytic bacteria [50,57,60], therefore explaining the low abundance of isolates with this feature. The association between an isolate’s ability to produce cellulase and its genus suggests that cellulase production may be an evolved feature for the endophytic lifestyle of strains belonging to specific genera.

As expected, only a small number of isolates are able to inhibit *Fusarium oxysporum* f. sp. ciceri growth and development through direct contact. Although other studies have reported the isolation of endophytic bacteria with antifungal activity, usually, the frequency of those bacteria is low or rare [61,62]. Nevertheless, their use as biocontrol agents has shown that these bacteria are able to suppress pathogens and promote plant growth [62,63].

The association between an isolate’s genus and its ability to produce ammonia, solubilize phosphate or synthesize IAA suggests that some plant growth-promoting traits may be species related. A similar pattern was observed with chickpea mesorhizobial isolates’ species cluster and their plant growth-promoting abilities [44].

Remarkably, almost all endophytic bacterial isolates characterized in the present study are tolerant to salinity although no association was found between an isolate’s tolerance to salt and the soil of origin. Similarly, a number of bacterial endophytes isolated from tomato grown in different soils also showed a high level of salt tolerance [42]. It is possible that endophytes require stress tolerance mechanisms to cope with the different stress conditions such as mineral content, availability of oxygen and pH variations, within plant tissues. Therefore, it is perhaps not surprising that salt tolerance is one of the multiple characteristics needed for the different strategies for interaction, lifestyle and survival inside of plant tissues. On the other hand, a significant relationship between an isolate’s tolerance to Mn and different soil treatments was observed. This result may be due to characteristics of the original soil, such as soil pH. In fact, a higher proportion of Mn-tolerant isolates was obtained from soils with a soil pH ≤ 6 while the Mn-sensitive isolates were mainly obtained from Monte da Pedra soil, with a soil pH of 7.74, and from Herdade da Mitra soil amended with dolomitic limestone, which is known for increasing the soil pH [64]. Since the availability of Mn in soils depends on the soil pH, where high soil pH reduces Mn availability and low soil pH increase Mn availability even to the point of toxicity, it may be speculated that soils with low pH may act as a selective pressure based on bacterial adaptive mechanisms, such as the tolerance to specific metals. This is evident, in particular, when increasing the pH in Herdade da Mitra soil with dolomitic limestone, a higher proportion of Mn-sensitive isolates were found in limestone-amended soils. Therefore, it can be assumed that changes in soil pH influenced the diversity and composition of the bacterial community in the soil, contributing to the growth of specific taxa, especially the Mn-sensitive bacteria, allowing them to compete and colonize the interior of plant root tissues. Together, these results are in agreement with previous studies [44,65,66] that suggest that an isolate’s tolerance is related to the original soil or to the isolates’s affiliation. Moreover, the powerful effect of the soil on the ecology of the endophytic bacterial communities has been noted in earlier studies [67,68,69], which led to the general assumption that most endophytes originate from soil. Yet, other studies show evidence that plant endophytic compartments tend to harbor similar microbial communities among different sites [70] and those endophytic communities are distinct assemblages rather than opportunistic subsets of the rhizosphere [71]. These differences found between microbial communities among different sites may be a result of the specific characteristics of those soils, such as pH, as observed herein.

Similarly, agricultural practices, like seed inoculation with rhizobium, may induce differences in the endophytic bacteria community in plant roots. In a study conducted by Zhang et al. [72], the diversity of soybean root endophytic bacteria was significantly affected by the three factors analyzed, namely, the plant growth stage, intercropping with maize, and rhizobial inoculation, though the latter was the factor that least affected the endophytic bacterial community structure. Our data indicate that rhizobial inoculation induced significant differences in the multifunctionality of the bacterial endophytes from inoculated chickpea plants. This result may be the explanation for the results obtained earlier. In addition, it is possible that the endophytic bacterial communities present in the formed root nodules were also considerably changed with rhizobia inoculation, as previously observed by Lu et al. [73].

## 4. Materials and Methods

### 4.1. Soil Samples and Plant Material

**Figure 7:**
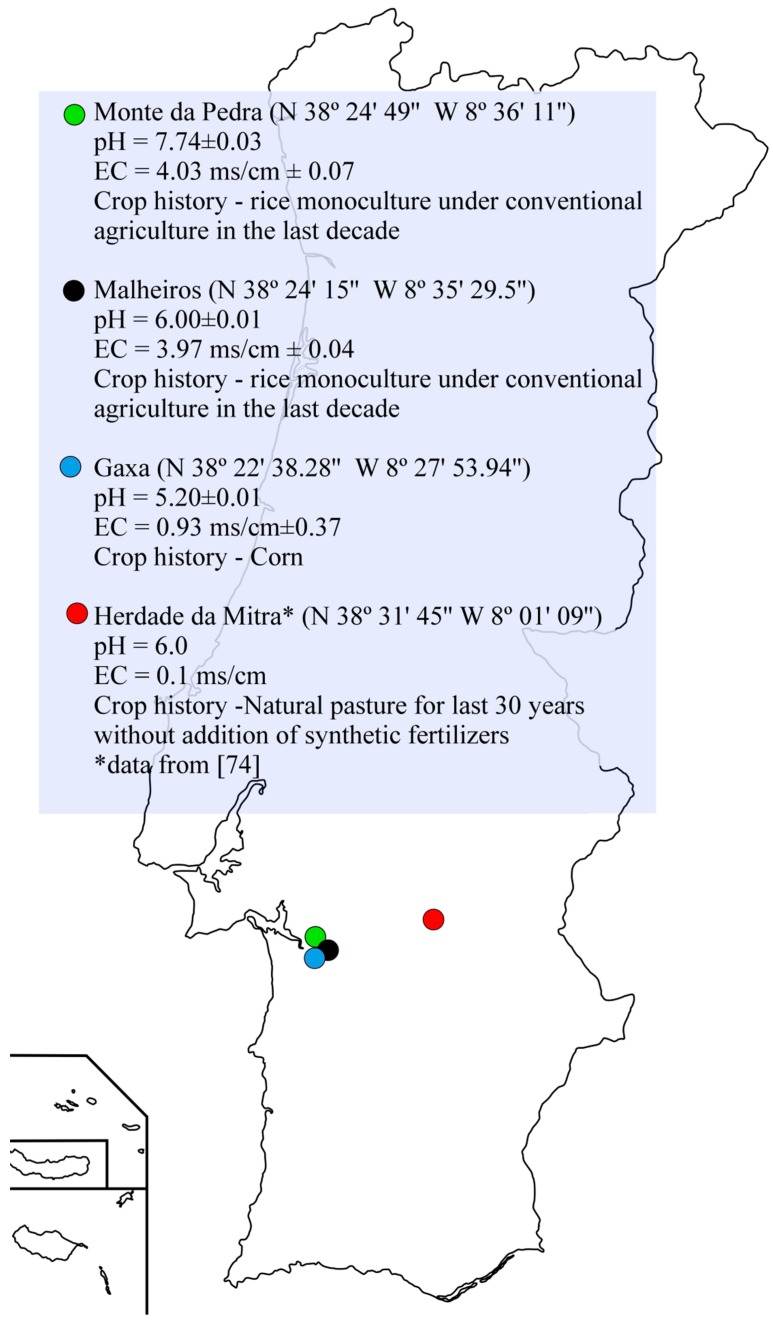
Map of Portugal with the four harvesting sites marked: Monte da Pedra, Malheiros, Gaxa and Herdade da Mitra. The pH and electrical conductivity (EC), geographical coordinates and crop history of each soil sample are indicated in the blue box.

**Figure 8:**
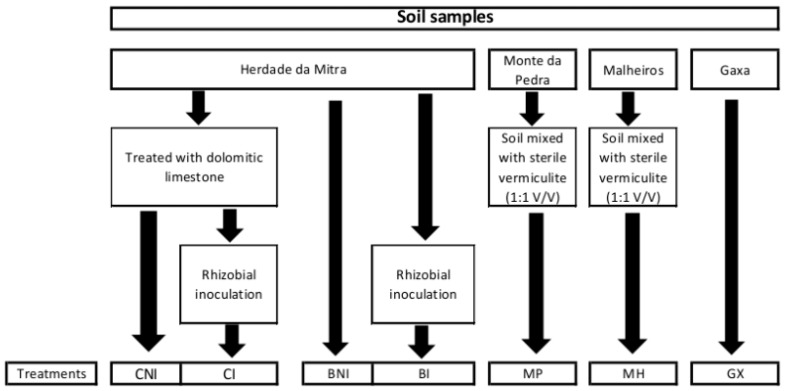
Schematic representation of the experimental design used in this study.

Soil samples collected from four different locations in Portugal were used in this study to isolate non-rhizobial endophytic bacteria using chickpea as trap plants (Figure 7). Herdade da Mitra sample is a Cambisoil derived from granites collected from a field located at the University of Évora, Portugal. Analytical characteristics of this soil were previously reported [74]. Although some reports using this soil showed that constraints to plant growth are mainly attributed to manganese toxicity [75,76,77], it possesses high microbial diversity [78]. Since this soil is well-characterized, it was chosen to evaluate the hypotheses that stress and rhizobia inoculation influence the diversity and functionality of endophytic bacterial communities. For that, “Herdade da Mitra” soil subsamples with and without dolomitic limestone (to relieve the manganese toxicity present in this soil) were used and a subsample of those were inoculated with the chickpea microsymbiont, *Mesorhizobium* sp. strain LMS-1 [79], as previously described [80]. Dolomitic limestone was applied at a rate of 1000 mg·kg^−1^ of soil according to a previous study [81]. To test the hypothesis that soil influences the endophytic bacterial communities, three soil samples from Alcaçer do Sal region, Portugal, were collected and their pH and electrical conductivity values were determined (Figure 7). Due to the high salinity level (based on electrical conductivities values) of the Monte da Pedra and Malheiros sites, these soil samples were mixed with sterile vermiculite (1:1 *v*/*v*) immediately before filling the pots. A total of seven treatments were considered in this study (details in Figure 8).

Chickpea seeds (*Cicer arietinum* L. cultivar Chk 3226) were surface sterilized and pre-germinated for 48 h as previously described [80]. After germination, the seeds were transferred to the pots previously filled with an unsterilized soil sample or a mixture of soil with vermiculite. Five chickpea plants were used per treatment. The pot experiments were grown under greenhouse conditions (where the maximum temperature allowed was set to 30 °C; and with 12.5 to 14.0 daylight hours from the beginning to the end of the 5-week plant trial), and watered whenever necessary with sterile distilled water.

### 4.2. Isolation of Bacterial Endophytes

At the end of the pot experiment, plants were harvested in the laboratory and were individually washed in tap water to remove any adhering soil particles. The visible root nodules were removed from the roots with a sterile clamp and the roots were subsequently surface sterilized according to Rashid et al. [42]. A 100-µL aliquot of the last sterile water rinse was platted onto Tryptic Soy agar (TSA; Merck) plates to assess the efficiency of the sterilization process. Only root material that showed a complete absence of any bacterial growth after 48 h at 28 °C was considered for further analysis. From these, three chickpea roots per treatment were used for bacterial endophyte isolation.

Isolation of bacterial endophytes was performed as previously described [42], using serial dilutions with 3× Ringer’s solution [82] to plate onto different media, namely, TSA (Merck), Luria agar (15 g·L^−1^ Agar; 10 g·L^−1^ Tryptone; 10 g·L^−1^ NaCl; 5 g·L^−1^ Yeast Extract), and King’s B agar [42]. After incubation at 25 °C for 72 h, colonies with different morphologies (based on size, shape, and color) were picked and sub-cultured separately [83]. Sub-culturing was performed 2-3 times until a pure culture was obtained and used for further analyses.

After isolation, a total of 59 bacterial endophyte strains were obtained (Table 1) and they were preserved in 30% glycerol at −80 °C. These strains were routinely grown in TSB (Merck) or in M9 minimal medium [84] when necessary.

### 4.3. Identification and Phylogenetic Analysis of Endophytic Bacteria

To extract the total genomic DNA from the endophytic bacteria, the bacterial cells of each isolate were collected in tubes containing 50 µL of lysis buffer (0.05 M NaOH and 0.1% SDS), subjected to 100 °C for 15 min and centrifuged at 13,000 rpm for 10 min. A 10-µL aliquot of the upper fraction was transferred to 90 µL of ultrapure sterile water.

Amplification of the 16S rRNA gene for each isolate was performed using the set of primers Y1 and Y3 [85]. The PCR reaction (50 µL) was prepared as follows: 1X reaction Buffer, 0.5 mM MgCl_2_, 0.2 mM of each dNTP, 10 pmol of each primer, 1 µL DNA (±1–10 ng) and 1.25 U DreamTaq DNA polymerase (Thermo Fisher Scientific Inc., USA). The amplification program used was: 5 min at 95 °C for initial denaturation; 35 cycles of 1 min at 95 °C; 1 min at 62 °C and 2 min at 72 °C, and a final extension step at 72 °C for 7 min. The PCR products were purified using a DNA Clean & Concentrator-5 Kit (ZymoResearch, Irvine, CA, USA) according to the manufacturer’s instructions, and subsequently sequenced by Macrogen Inc. (Seoul, Korea) using the universal primer 1100R (5’- GGGTTGCGCTCGTTG-3’) [86]. The obtained sequences were compared with those from the GenBank public database and the EzBioCloud database [87]. MEGA7 software [88] was used to align the 16S rRNA gene fragment (~650-750 bp) sequences using the ClustalW software [89] and to infer the molecular phylogeny by the Neighbor-Joining method [90] based on a distance matrix with the distance correction calculated by Kimura’s two-parameter model [21]. The robustness of the phylogenetic tree was evaluated by bootstrap analysis of 1,000 resamplings. The partial 16S rRNA nucleotide gene sequences obtained in this study have been deposited in the NCBI GenBank database under the accession numbers MH055461 to MH055519.

### 4.4. Screening and Identification of Cellulase Producers

The screening for cellulase-producing endophytic bacteria was done on carboxymethylcellulose (CMC) agar plates according to Kasana et al. [91]. The cellulase activity was estimated by measuring the zone of clearance around each colony and comparison of the size of this zone with the colony diameter. The presence of a zone of clearance around a colony was considered as positive for cellulase production. According to the zone of clearance, four different levels of cellulase activity were observed: (0 mm) no production or activity; (>0 mm and ≤5 mm) low production; (>5 mm and ≤10 mm) high production; (>10 mm) very high production.

### 4.5. Plant Growth-Promoting Properties of Bacterial Endophytes

The ability of the bacterial endophyte isolates to produce ammonia was tested accordingly to Marques et al. [92]. After addition of Nessler’s reagent, the development of a faint yellow color was considered as a small amount of ammonia produced whereas a deep yellow to brownish color indicated a large amount of ammonia production.

To evaluate the ability of the bacterial endophyte isolates to solubilize phosphate, the isolates were grown on Pikovskaya’s medium plates according to de Freitas et al. [93], for 7–10 days at 30 °C. A zone of clearance around the colonies was considered positive for phosphate solubilization.

To detect the ability of bacterial endophyte isolates to produce and secrete siderophores, 10 µL of each bacterial isolate from a culture grown for 24 h in TSB medium was spotted onto a CAS agar plate [94] in triplicate and incubated at 30 °C for 7–10 days. A color change of the CAS reagent from blue to orange was considered as positive for siderophore production.

The ability of bacterial endophyte isolates to produce indoleacetic acid (IAA) was measured as described by Brígido et al. [44]. According to the amount of IAA produced, three distinct levels of IAA production: no or low production (<20 µg·mL^−1^), medium production (between 20 and 50 µg·mL^−1^), and high production (>50 µg·mL^−1^) were considered.

### 4.6. In vitro Screening for Antagonistic Activity

The fungal agent used in this study was chosen based on its high pathogenic ability to cause wilt disease in chickpea plants. This pathogenic agent was isolated from diseased chickpea roots and sub-cultured in potato dextrose agar (PDA) until it was obtained in pure culture. Based on its 25S rRNA gene sequence, the fungal agent is closely related to *Fusarium oxysporum* f. sp. ciceri (99.8% identity) (*data not shown*).

The antifungal activity of each bacterial strain was determined by growing each of the bacterial strains together with the above-mentioned disease-causing fungal species. Briefly, 10 µL of bacterial culture grown in liquid M9 minimal medium was spotted in triplicate onto the margins of a PDA plate. Then, a 5-mm diameter piece of agar from a 7-day-old PDA plate of an overgrown culture of the fungal agent was placed in the center of the Petri plate. PDA plates inoculated only with the fungal agent were used as negative controls. Three independent experiments with each bacterial isolate were performed. The PDA plates were incubated at room temperature for 7 days. Inhibition of the mycelium development was considered positive for antifungal activity while no mycelium inhibition was considered negative.

### 4.7. Manganese and Salt Tolerance

The evaluation of bacterial endophyte isolates’ tolerance to salt and Mn was based on their growth in 96-well microtiter plates filled with 200 µL per well of M9 minimal medium supplemented with MnSO_4_ at final concentrations 0.1, 0.5, 1, 2.5, 5, 10, 20 mM for manganese tolerance and 0%, 1%, 2.5%, 5%, 10% of NaCl for salt tolerance. For each isolate, 20 µL of an initial inoculum with an OD_565nm_ = 0.05 was added into the 96 wells of the microtiter plate. Wells with non-inoculated medium served as a blank. The microtiter plates were incubated under agitation at 30 °C for 2 days. After incubation, the microtiter plates were read by spectrophotometry at OD_565nm_ using a microtiter plate reader (Multiskan spectrum, Thermo Scientific, Waltham, MA, USA.). The maximum tolerated concentration for the bacterial endophyte isolates in each stress condition was considered to be the previous concentration to that in which the isolates showed no growth.

### 4.8. Statistical Analysis

Statistical analyses were performed using SPSS 21.0 software (SPSS Inc., Chicago, IL, USA). Distributions of continuous samples were submitted to the one-sample Kolmogorov-Smirnov test to evaluate the goodness of fit of data to the normal distribution. The relationship between continuous dependent variables and categorical independent variables was explored with the Kruskal-Wallis one-way nonparametric analysis of variance. Relationships between categorical variables were determined using the chi-square test of association. Results are presented as the test statistic (χ^2^), degrees of freedom (*d.f.*), and probability of equal or greater deviation (*P*). When categorical variables had low frequencies (n < 5), the chi-square test of association was replaced by Fisher’s exact test [95]. To detect structure in the relationships between categorical variables, the correspondence analysis (CA) was conducted as an exploratory data analysis technique. Non-parametric correlations between continuous variables were determined using Spearman’s rank order correlation coefficient. The statistical differences (*P* < 0.05) of the proportions of nominal variables between two independent groups were examined through Fisher’s exact test.

## 5. Conclusions

Endophytic bacteria associated with chickpea plants possess multiple traits for plant growth promotion as well as tolerance to high concentration of manganese and NaCl, which may be important features in promoting legume growth under marginal conditions. Moreover, several plant growth-promoting traits in chickpea endophytic bacteria appear to be genus-specific while tolerance to manganese seems to be associated with the soil origin. Although preliminary, this study suggests that different variables shape the functionality of endophytic bacterial communities; these prominently include the soil origin (including aboveground diversity) and rhizobial inoculation. Nevertheless, additional studies using independent cultivation methods would contribute to determine, in greater depth, the effects of different environmental factors on endophytic bacterial communities and the *Cicer arietinum* microbiome. The understanding of the effects of environmental conditions on soil microbe functional diversity is important, together with inoculation, to capitalize the benefits of beneficial bacteria in sustainable crop production. The present study contributes to identify variables that have impact on functional diversity of endophytic bacteria in chickpea.

## Figures and Tables

**Table 1:** List of the endophytic bacteria isolates obtained from each treatment.

Soil Sample/Treatment		Isolates
**Herdade da Mitra soil without dolomitic limestome amendment (B)**	Inoculated with rhizobia (BI)	BI-1; BI-2; BI-3; BI-4; BI-5; BI-6
	Not inoculated with rhizobia (BNI)	BNI-1; BNI-2; BNI-3; BNI-4; BNI-5; BNI-6; BNI-8; BNI-9; BNI-10; BNI-11; BNI-12
**Herdade da Mitra soil amended with dolomitic limestone (C)**	Inoculated with rhizobia (CI)	CI-1; CI-2; CI-3; CI-4; CI-5; CI-6; CI-7; CI-8; CI-9; CI-10; CI-11; CI-12
	Not inoculated with rhizobia (CNI)	CNI-1; CNI-2; CNI-3; CNI-4; CNI-5; CNI-6; CNI-7; CNI-8; CNI-9; CNI-10
**Malheiros soil (MH)**		MH-1; MH-2; MH-3; MH-4; MH-5; MH-6
**Monte da Pedra soil (MP)**		MP-1; MP-2; MP-3; MP-4; MP-5; MP-6; MP-7; MP-8
**Gaxa soil (GX)**		GX-1; GX-2; GX-3; GX-4; GX-5; GX-6

**Table 2:** Taxonomic identification of cultured endophytic bacteria based on sequencing of the partial 16S rDNA gene sequence obtained from each treatment: A black box indicates presence of that genus. Herdade da Mitra soil (BNI) with rhizobial inoculation (BI); Herdade da Mitra soil amended with dolomitic limestone (CNI) with rhizobial inoculation (CI); Malheiros soil (MH); Monte da Pedra soil (MP); Gaxa soil (GX). More details on bacterial isolates are found in Additional file: Table S1.

Isolate Genus	Treatments						
	BNI	BI	CNI	CI	MH	MP	GX
*Leifsonia*							
*Microbacterium*							
*Bacillus*							
*Paenibacillus*							
*Staphylococcus*							
*Stenotrophomonas*							
*Pseudomonas*							
*Pantoea*							
*Enterobacter*							
*Klebsiella*							
*Kosakonia*							
*Rhizobium*							
Total genera	6	4	4	6	5	6	4

## Supplementary Materials

The following are available online at http://www.mdpi.com/2223-7747/8/2/42/s1, Table S1: All results obtained for each bacterial endophyte.
