# Supplementary material for: Diversity and Functionality of Culturable Endophytic Bacterial Communities in Chickpea Plants
Source: Plants (Basel). 2019 Feb 14;8(2):42. doi: 10.3390/plants8020042 (PMC6409739; doi:10.3390/plants8020042)
Supplement: Supplementary file 1 [file plants-08-00042-s001.pdf]

Table S1. Results obtained for cellulase activity, IAA synthesis, siderophore and ammonia production, phosphate solubilization and antifungal activity. (−) no production or activity; (+) low production; (++) high production; (+++) very high production; nd- not determined. Maximum salt and manganese concentration tolerated and genus based on partial 16S rRNA gene sequence for each endophytic bacterial strain.

Strain	IAA production [µg/ml]	Cellulase production	Siderophore production	Phosphate solubilization	Ammonia production	Antifungal activity	Maximun NaCl concentration tolerated [%]	Maximun Mn concentration tolerated [mM]	Genus / Species affiliation	GenBank accession number
**BI-1**	57.0	−	no	no	yes	no	7.5	5.0	*Pantoea* sp.	MH055461
**BI-2**	2.8	++	yes	yes	yes	no	5.0	2.5	*Bacillus* sp.	MH055462
**BI-3**	1.6	+++	yes	yes	yes	no	10.0	2.5	*Pseudomonas* sp.	MH055463
**BI-4**	82.2	−	yes	no	yes	no	2.5	2.5	*Bacillus acidiceler*	MH055464
**BI-5**	88.9	++	no	yes	no	no	5.0	5.0	*Enterobacter* sp.	MH055465
**BI-6**	7.2	++	yes	no	yes	no	2.5	2.5	*Bacillus* sp.	MH055466
**BNI-1**	80.1	−	no	no	no	no	7.5	2.5	*Enterobacter* sp.	MH055467
**BNI-2**	46.5	−	no	no	no	no	7.5	2.5	*Pantoea* sp.	MH055468
**BNI-3**	0.1	++	no	no	no	no	2.5	2.5	*Stenotrophomonas* sp.	MH055469
**BNI-4**	44.9	−	no	no	yes	no	5.0	1.0	*Enterobacter* sp.	MH055470
**BNI-5**	16.6	−	yes	no	no	yes	5.0	0.0	*Paenibacillus* sp.	MH055471
**BNI-6**	2.6	−	no	no	yes	no	10.0	0.0	*Pseudomonas* sp.	MH055472
**BNI-8**	82.6	−	yes	no	yes	no	5.0	2.5	*Enterobacter* sp.	MH055473
**BNI-9**	73.4	−	no	no	yes	no	7.5	2.5	*Enterobacter* sp.	MH055474
**BNI-10**	0.0	−	no	no	no	no	2.5	0.1	*Bacillus* sp.	MH055475
**BNI-11**	0.0	−	no	no	yes	no	10.0	0.5	*Bacillus* sp.	MH055476
**BNI-12**	70.2	−	no	no	yes	nd	5.0	0.5	*Enterobacter* sp.	MH055477
**CI-1**	88.0	−	no	no	no	no	5.0	0.5	*Enterobacter ludwigii*	MH055478
**CI-2**	2.1	+	yes	no	yes	yes	2.5	0.1	*Pseudomonas protegens*	MH055479
**CI-3**	4.3	+	no	yes	yes	no	7.5	0.5	*Klebsiella* sp.	MH055480

**CI-4**	87.4	−	yes	no	no	no	5.0	0.5	*Enterobacter* sp.	MH055481
**CI-5**	50.8	−	no	yes	yes	no	7.5	0.5	*Enterobacter ludwigii*	MH055482
**CI-6**	88.2	−	no	no	yes	no	2.5	2.5	*Enterobacter* sp.	MH055483
**CI-7**	21.3	+	no	no	yes	no	2.5	0.25	*Stenotrophomonas* sp.	MH055484
**CI-8**	64.1	−	no	yes	yes	no	5.0	0.5	*Enterobacter ludwigii*	MH055485
**CI-9**	86.6	−	no	yes	no	no	5.0	5.0	*Enterobacter ludwigii*	MH055486
**CI-10**	5.8	−	yes	yes	no	no	7.5	5.0	*Staphylococcus saprophyticus* subsp. *bovis*	MH055487
**CI-11**	0.6	−	no	no	yes	no	1.0	0.25	*Pseudomonas* sp.	MH055488
**CI-12**	0.0	+	no	no	yes	no	2.5	0.1	*Microbacterium* sp.	MH055489
**CNI-1**	9.0	+	no	no	yes	no	2.5	0.5	*Pseudomonas* sp.	MH055490
**CNI-2**	0.1	−	no	no	no	yes	10.0	0.25	*Stenotrophomonas* sp.	MH055491
**CNI-3**	4.8	++	yes	no	yes	yes	2.5	2.5	*Pseudomonas koreensis*	MH055492
**CNI-4**	4.7	−	yes	no	yes	yes	10.0	0.1	*Pseudomonas koreensis*	MH055493
**CNI-5**	6.4	++	no	no	yes	nd	2.5	0.0	*Pseudomonas* sp.	MH055494
**CNI-6**	0.4	−	yes	no	yes	no	5.0	5.0	*Enterobacter* sp.	MH055495
**CNI-7**	0.4	−	no	no	yes	no	7.5	0.1	*Pseudomonas paralactis*	MH055496
**CNI-8**	8.3	+	no	no	yes	no	2.5	0.1	*Pseudomonas* sp.	MH055497
**CNI-9**	34.3	++	yes	no	yes	no	10.0	0.0	*Microbacterium* sp.	MH055498
**CNI-10**	65.7	−	yes	no	yes	nd	5.0	5.0	*Enterobacter* sp.	MH055499

**GX1**	6.7	−	nd	nd	no	no	0.0	5.0	*Paenibacillus* sp.	MH055500
**GX2**	1.1	−	no	no	yes	no	5.0	0.5	*Pantoea* sp.	MH055501
**GX3**	0.0	+++	yes	no	no	no	5.0	2.5	*Paenibacillus* sp.	MH055502
**GX4**	0.1	−	no	yes	no	no	7.5	0.5	*Leifsonia soli*	MH055503
**GX5**	3.5	−	nd	nd	yes	no	0.0	0.5	*Bacillus* sp.	MH055504
**GX6**	2.2	−	no	no	yes	no	10.0	5.0	*Bacillus* sp.	MH055505
**MH1**	29.4	+	yes	no	yes	no	7.5	0.5	*Pantoea* sp.	MH055506
**MH2**	1.0	−	no	no	yes	yes	2.5	0.5	*Pseudomonas protegens*	MH055507
**MH3**	78.9	−	yes	no	yes	no	7.5	0.5	*Enterobacter* sp.	MH055508
**MH4**	4.1	−	no	no	yes	yes	7.5	5.0	*Bacillus* sp.	MH055509
**MH5**	45.7	+	yes	yes	yes	no	5.0	1.0	*Kosakonia* sp.	MH055510
**MH6**	6.3	−	no	no	yes	no	5.0	0.5	*Pseudomonas* sp.	MH055511
**MP1**	14.3	−	yes	no	no	no	2.5	0.5	*Rhizobium* sp.	MH055512
**MP2**	5.4	−	no	no	yes	no	10.0	0.25	*Stenotrophomonas* sp.	MH055513
**MP3**	2.6	+++	no	no	no	no	2.5	0.1	*Paenibacillus* sp.	MH055514
**MP4**	88.1	−	no	no	yes	no	5.0	0.5	*Enterobacter ludwigii*	MH055515
**MP5**	3.7	++	no	no	no	no	2.5	2.5	*Bacillus* sp.	MH055516
**MP6**	4.9	−	no	no	yes	no	2.5	0.25	*Stenotrophomonas* sp.	MH055517
**MP7**	7.0	+	no	no	no	no	2.5	0.25	*Pseudomonas brassicacearum* subsp. *neoaurantiaca*	MH055518
**MP8**	1.0	−	no	no	yes	no	2.5	2.5	*Stenotrophomonas* sp.	MH055519
